# Endoscopic advances in gastroenterology

**DOI:** 10.1093/gastro/goad046

**Published:** 2023-08-10

**Authors:** Ashley L Faulx, Amitabh Chak

**Affiliations:** Department of Gastroenterology and Hepatology, Louis Stokes VA Medical Center, Cleveland, OH, USA; Division of Gastroenterology and Hepatology, University Hospitals Cleveland Medical Center, Case Western Reserve University, Cleveland, OH, USA; Division of Gastroenterology and Hepatology, University Hospitals Cleveland Medical Center, Case Western Reserve University, Cleveland, OH, USA

This Special Issue of *Gastroenterology Report* is dedicated to endoscopic advances that will have meaningful impacts on clinical practice. A panel of internationally recognized endoscopists have written informative updates covering their expertise.

Upper gastrointestinal bleeding continues to be associated with significant morbidity and mortality, although hospitalization for peptic ulcer disease—the most common etiology of upper gastrointestinal bleeding—has decreased by 30% over the past decade, while increases have been seen for other etiologies such as Dieulafoy lesions, cancer, and angioectasias. In this issue, Drs Alali and Barkun [1] discussed the causes of upper gastrointestinal bleeding and the various risk-assessment tools to aid the clinician in risk stratification of patients. New advances such as hemostatic powders, over-the-scope clips, and the endoscopic Doppler probe were reviewed, and the best application of this new technology was defined.

Obesity is a worldwide epidemic, associated with a significant increase in all-cause and cardiovascular mortality, as well as diabetes, hypertension, and non-alcoholic fatty liver disease. Bariatric surgery has been the mainstay modality for patients with obesity, though <2% of eligible patients undergo the surgery. Endoscopic bariatric procedures are procedures indicated for patients with obesity who do not qualify for surgery or who choose a more minimally invasive approach to weight loss. Drs Schulman and Shenoy [2] discussed the various weight loss devices such as gastric balloons, the Transpyloric shuttle, aspiration tubes, duodenal sleeves, and small bowel magnets. They presented an in-depth discussion of endoscopic suturing techniques for weight loss, including sleeve gastroplasty and primary obesity surgery endoluminal. Endobariatrics is a rapidly growing field and may offer patients the advantages of surgical bypass using a minimally invasive approach.

International expert on colonoscopy and polypectomy, Dr Rex [3], discussed key quality indicators in colonoscopy, including the history of the quality movement and a discussion of methods of improving adenoma detection rates. Several techniques and technological advances now enable endoscopists to successfully remove larger polyps that were previously referred for surgery. To decide the proper course of therapy, it is critical for endoscopists to learn polyp characteristics that suggest the presence of malignancy. Dr Gao *et al.* [4] presented current techniques for polyp histology recognition as well as methods for large polyp endoscopic resection.

Endoscopic eradication has become standard of care for patients with dysplastic Barrett’s esophagus, as it has been shown to decrease the risk of progression to adenocarcinoma. In addition to laying out the indications for endoscopic eradication and discussion of the role of endoscopic ultrasound, Drs Vantanasiri and Iyer [5] updated readers on the newest modalities for endoscopic resection and ablation of Barrett’s esophagus.

Endoscopic ultrasound has evolved from a diagnostic technique to a sophisticated interventional tool since its inception as an imaging modality >40 years ago. Endoscopic ultrasound-guided techniques now include gall bladder and pancreatic fluid drainage procedures, injection therapy, enteral anastomosis formation, and a variety of hepatology applications. Dr Radlinski *et al*. [6] described these novel techniques that are rapidly being implemented into clinical practice. Although some of these techniques are limited to specialized centers, development of new tools will help wider adoption in the near future.

Third-space endoscopy, or endoscopy that accesses the submucosa, began with per-oral endoscopic myotomy (POEM). This led to an appreciation that mucosal incision with submucosal tunneling could have many other applications. Dr Hayat *et al*. [7] described a variety of third-space endoscopy techniques, including esophageal POEM, Zenker’s POEM, and gastric POEM, as well as the use of submucosal tunneling to resect sub-epithelial lesions. These techniques continue to evolve. With the exception of POEM, which is now the standard of care for achalasia, data on long-term outcomes for these other techniques are needed.

We hope the readers will enjoy reading this Special Issue as much as we have enjoyed putting it together.
